# Design and fabrication of far ultraviolet filters based on π-multilayer technology in high-*k* materials

**DOI:** 10.1038/srep08503

**Published:** 2015-02-17

**Authors:** Xiao-Dong Wang, Bo Chen, Hai-Feng Wang, Fei He, Xin Zheng, Ling-Ping He, Bin Chen, Shi-Jie Liu, Zhong-Xu Cui, Xiao-Hu Yang, Yun-Peng Li

**Affiliations:** 1 Changchun Institute of Optics, Fine Mechanics and Physics, Chinese Academy of Sciences, Changchun 130033, China

## Abstract

Application of π-multilayer technology is extended to high extinction coefficient materials, which is introduced into metal-dielectric filter design. Metal materials often have high extinction coefficients in far ultraviolet (FUV) region, so optical thickness of metal materials should be smaller than that of the dielectric material. A broadband FUV filter of 9-layer non-periodic Al/MgF_2_ multilayer was successfully designed and fabricated and it shows high reflectance in 140–180 nm, suppressed reflectance in 120–137 nm and 181–220 nm.

In research for terrestrial aurora emission in FUV, narrowband and broadband filters are widely employed because unwanted line emission should be blocked to ensure high spectral purity in the working wavelength^1^,^2^,^3^. Zukic and Torr did many pioneering jobs about FUV filters, and they proposed π-multilayer technology to design FUV filters^4^,^5^,^6^. FUV filters designed and fabricated by them were successfully utilized in ultraviolet imager for the International Solar–Terrestrial Physics mission^1^,^2^ and IMAGE satellite^3^.

In optical thin film design for FUV spectral range, LaF_3_, MgF_2_ and Al are widely used due to their excellent optical properties. MgF_2_ is transparent at wavelength longer than 115 nm; Al is the material with highest reflection in the vacuum ultraviolet down to 83 nm. Al/MgF_2_ and LaF_3_/MgF_2_ are two commonly employed material pairs in FUV wavelength range, and they are deposited by thermal evaporation method. Al/MgF_2_ films were used as reflectance mirrors^7^,^8^,^9^,^10^,^11^, transmittance filters^12^,^13^,^14^,^15^,^16^ and reflectance filters^17^. In the beginning, MgF_2_ was just used as a protective layer in order to avoid oxidation of Al. MgF_2_-overcoated Al (two layers) mirrors have been extensively studied^7^,^8^,^9^,^10^,^11^. Malherbe deposited a double half-wave filter type of Al/MgF_2_ for Lyman-*a* line (121.6 nm) with a full width measured at half of the transmittance maximum (FWHM) of 9.0 nm^12^. Bates and Bradley prepared interference filters of Al/MgF_2_ in the wavelength range of 170 to 240 nm, and the peak transmission is 25% with a band pass <30.0 nm^13^. Spiller fabricated Al/MgF_2_ transmittance filters with different values of FWHM, and they ascribed the discrepancies between theoretical and experimental results to a coupling of light into the surface Plasmon of Al via a surface roughness of the MgF_2_ layers^14^. Transmittance filters of Al/MgF_2_ mentioned above were all designed based on the theory of Fabry-Perot interference filters. To authors' knowledge, reflectance filters of Al/MgF_2_ were only studied by Osantowski and Toft^17^. They prepared broadband ultraviolet reflectance filters of Al/MgF_2_ with peak reflectance at 185.0, 230.0, and 260.0 nm^17^, and the FWHM can be up to be 120 nm. However, the detailed design was not provided. Reflectance filters made of Al/MgF_2_ have larger values of FWHM as compared to LaF_3_/MgF_2_^4^,^17^.

Besides filters in FUV deposited by physical vapor deposition method (thermal evaporation, magnetron sputtering), recently, some ultraviolet mirrors deposited by sol-gel were reported^18^,^19^,^20^. ZrO_2_/SiO_2_ nanoparticle films were prepared to selectively block ultraviolet wavelength ranges based on interference effects, and they can be self-standing, flexible, transferable if a polymer was infiltrated in the pore network of films^18^. Fe^3+^ doped Mg/Al coatings were prepared by a facile solvothermal method, and their ultraviolet-absorption was efficiently tuned by Fe^3+^ to cover the entire ultraviolet range^19^. Perforated multilayers were generated by collective osmotic shock to reflect selectively ultraviolet radiation^20^. Films prepared by sol-gel demonstrate some better properties as compared to physical vapor deposition. However, to our knowledge, there are no multilayers prepared by sol-gel at targeted wavelength of less than 200 nm, and few optical thin films prepared by sol-gel were employed in space exploration. Surface roughness of films has a great influence on optical performance of multilayers at targeted wavelength of less than 200 nm. Optical coatings in the space environment are exposed to high energy particulate, electromagnetic radiations, and large temperature excursions^21^, and they must have a stable structure. Great improvements in optical coatings deposited by sol-gel should be made, such as layer thickness control, surface roughness, and structural stability.

A special multilayer mirror was employed in the FUV Wideband Imaging Camera installed in IMAGE satellite, and it has working wavelength region from 140–190 nm, also has low reflectance in the visible and near ultraviolet region^3^. However, they did not tell us what the specific material pair they used was. Similar broadband filters are also in great demand in an Imager installed in FengYun III, which will be launched in 2015. we want to yield spectra emission of N_2_ Lyman-Birge-Hopfield Bands (140–180 nm), and suppress the two oxygen emissions at 130.4 and 135.6 nm, hydrogen Lyman-α line at 121.6 nm, and contamination signals from the wavelength region longer than 180 nm by virtue of this filter^2^. In this paper, a broadband filter of non-periodic Al/MgF_2_ was designed based on modified π-multilayer technology and fabricated.

## Theory and design

π-multilayer is defined as one has a periodic optical thickness of a half of reference wavelength, which can be described by equation (1)^4^:
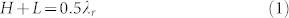
where *H* and *L* denote optical thickness of high- and low-index film materials, and *λ*_r_ is the reference wavelength. If *H* = *L* = 0.25*λ*_r_, that is a traditional quarter-wave (QW) multilayer. In FUV wavelength region, BaF_2_ and LaF_3_ are often employed to be high-index film materials, and MgF_2_ low-index one. The extinction coefficients of BaF_2_ and LaF_3_ are 100 times larger than that of MgF_2_. Zukic and Torr believed that if *H*/*L* < 1, absorption will be lower, hence filters with higher reflectance and a narrower FWHM can be obtained. The above description of π-multilayer technology is just a brief introduction, and the details can be found in Ref. 4.

It should be noted that Zukic and Torr just studied multi-dielectric components for the FUV, and π-multilayer technology can be modified to design metal-dielectric multilayer. In other words, Zukic and Torr just discussed the application of π-multilayer technology in low-absorption materials, and we want to extend the application of π-multilayer technology to high-absorption materials. Here, we choose Al/MgF_2_ to be a candidate material pair of a multilayer because besides LaF_3_/MgF_2_^4^,^22^, Al/MgF_2_ is another commonly used one in FUV region^7^,^8^,^9^,^10^,^11^,^12^,^13^,^14^,^15^,^16^,^17^.

**Figure 1:**
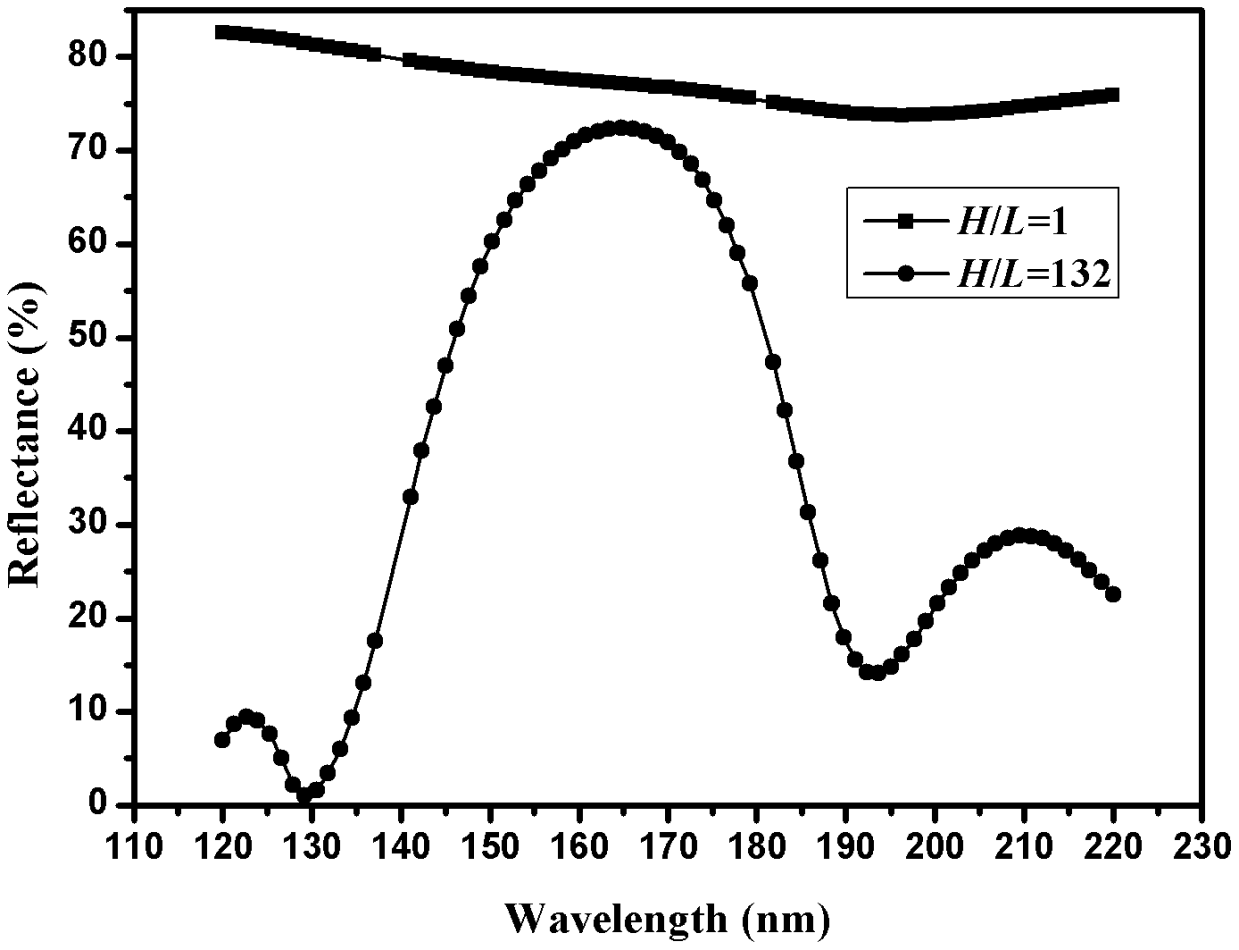
Theoretical reflectance of 9-layer periodic Al/MgF_2_ multilayer with an incident angle of 22° for *H*/*L* = 1, 132.

In this material pair, MgF_2_ is high-index material, and has a magnitude of 10^−4^ for extinction coefficient^5^; Al is low-index material, and has a magnitude of 10° for extinction coefficient^23^; fused silica is substrate material (thickness of 1.5 mm). The optical constant of MgF_2_ was derived from characterization of reflectance and transmittance of deposited 160-nm single MgF_2_ layer (by OptiLayer software^24^), and the optical constants of Al and fused silica substrate are cited from Center for Nanolithography Research, Rochester Institute of Technology. Our aim is to obtain high reflectance in the wavelength range of 140 nm to 180 nm, meanwhile, suppressed reflectance in 120–137 nm and 181–220 nm. As we all know, QW Al/MgF_2_ multilayer has enough high reflectance due to high reflectance of Al layer (Figure 1). Al film has lower refractive index with a magnitude of 10^−2^, and larger extinction coefficient with a magnitude of 10°. The reflectance of Al film can be calculated by equation (2) (the incident medium is air):
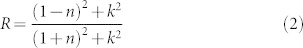
where *n* is refractive index, *k* is extinction coefficient. The reflectance of Al film is dependent on *k* because of *k* ≫ *n* for Al film. Al film still has high reflectance even if the optical thickness of Al layer is largely less than QW. This can be interpreted by a general rule that if any material gets to be a very good absorber at any frequency, the waves are strongly reflected at the surface and very little gets inside to be absorbed. More details about this rule can be found in Ref. 25.

Now our problem is not to try to enhance reflectance, but to suppress the reflectance in 120–137 nm and 181–220 nm at the expense of a slight drop of reflectance in 140–180 nm. In other words, we want to design a broadband filter instead of a reflectance mirror. Thus, inversely, we should keep *H*/*L* > 1. Figure 1 shows theoretical reflectance of 9-layer periodic Al/MgF_2_ multilayer for *H*/*L* = 1, 132, the reference wavelength is 160 nm, and the incident angle is 22°. For *H*/*L* = 1, that is QW multilayer, it has reflectance of 74–83% in 120–220 nm. For *H*/*L* = 132, it corresponds to optical thicknesses of high- and low-index film materials:
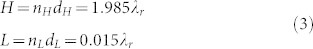
The 9-layer stack with this ratio has a filter feature, and the FWHM is 43 nm.

**Figure 2:**
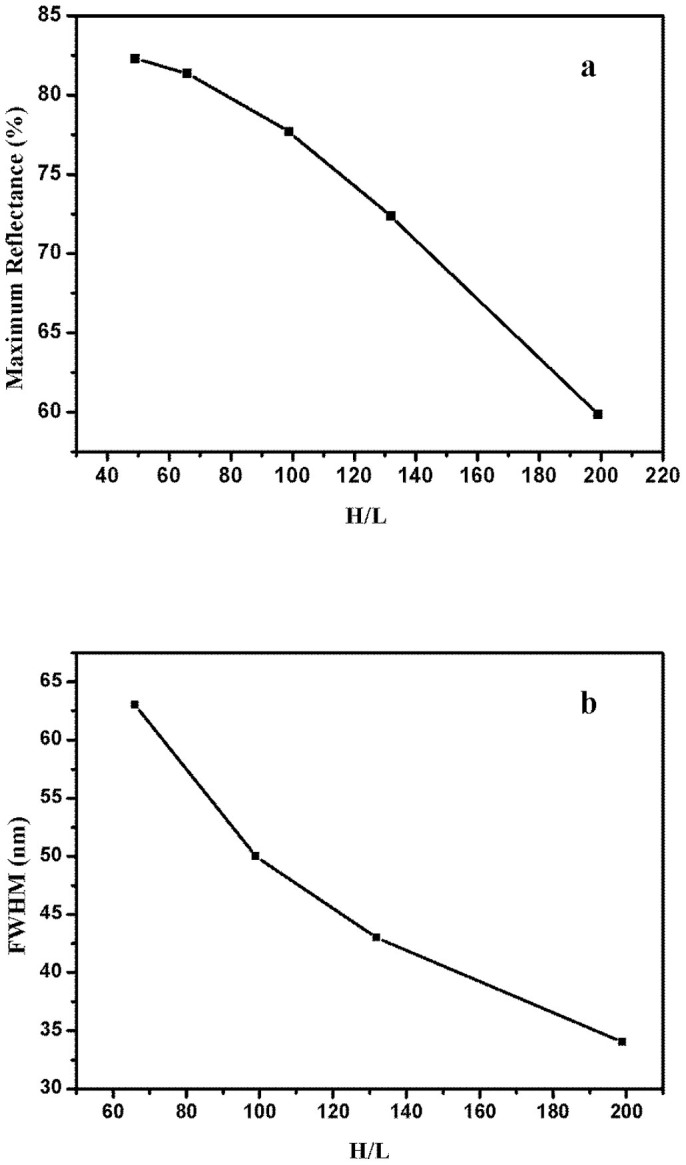
Calculated maximum reflectance (a) and FWHM (b) of 9-layer periodic Al/MgF_2_ multilayer as a function of the *H*/*L* ratio.

**Figure 3:**
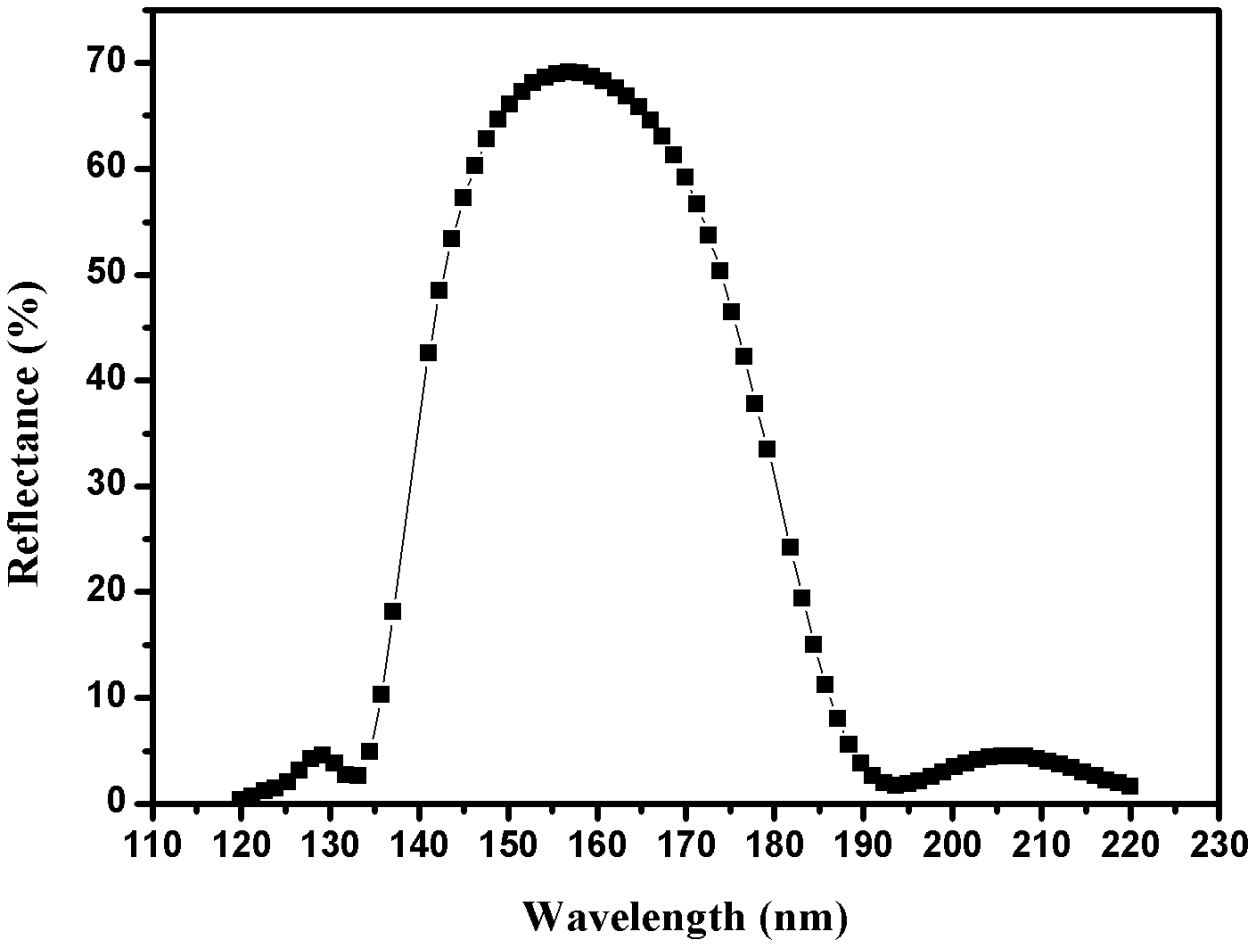
The refined, calculated result of 9-layer non-periodic Al/MgF_2_ multilayer with an incident angle of 22°.

Figure 2 demonstrates calculated maximum reflectance and FWHM of 9-layer Al/MgF_2_ multilayer as a function of the *H*/*L* ratio. The maximum reflectance and FWHM decrease with increasing of *H*/*L* ratio. The ratio of *H*/*L* can not be too big, and we must consider the thinnest feasible Al layer. As shown in Figure 2, according to our aim, 9-layer Al/MgF_2_ multilayer with *H*/*L* = 132 seems to be the most feasible choice. However, the suppression feature in longer wavelength is not so good. Thus, we use Refinement function (OptiLayer software^24^) to optimize the performance of 9-layer periodic Al/MgF_2_ broadband filter for *H*/*L* = 132. The refined, calculated result is shown in Figure 3, the FWHM is 40 nm, and this non-periodic filter has relatively better reflectance suppression in 120–137 nm and 181–220 nm, which meets our requirements. The optical thickness of the filter is 402.1 nm. The physical thickness of Al layer varies from 4.0–10.0 nm.

## Results

**Figure 4:**
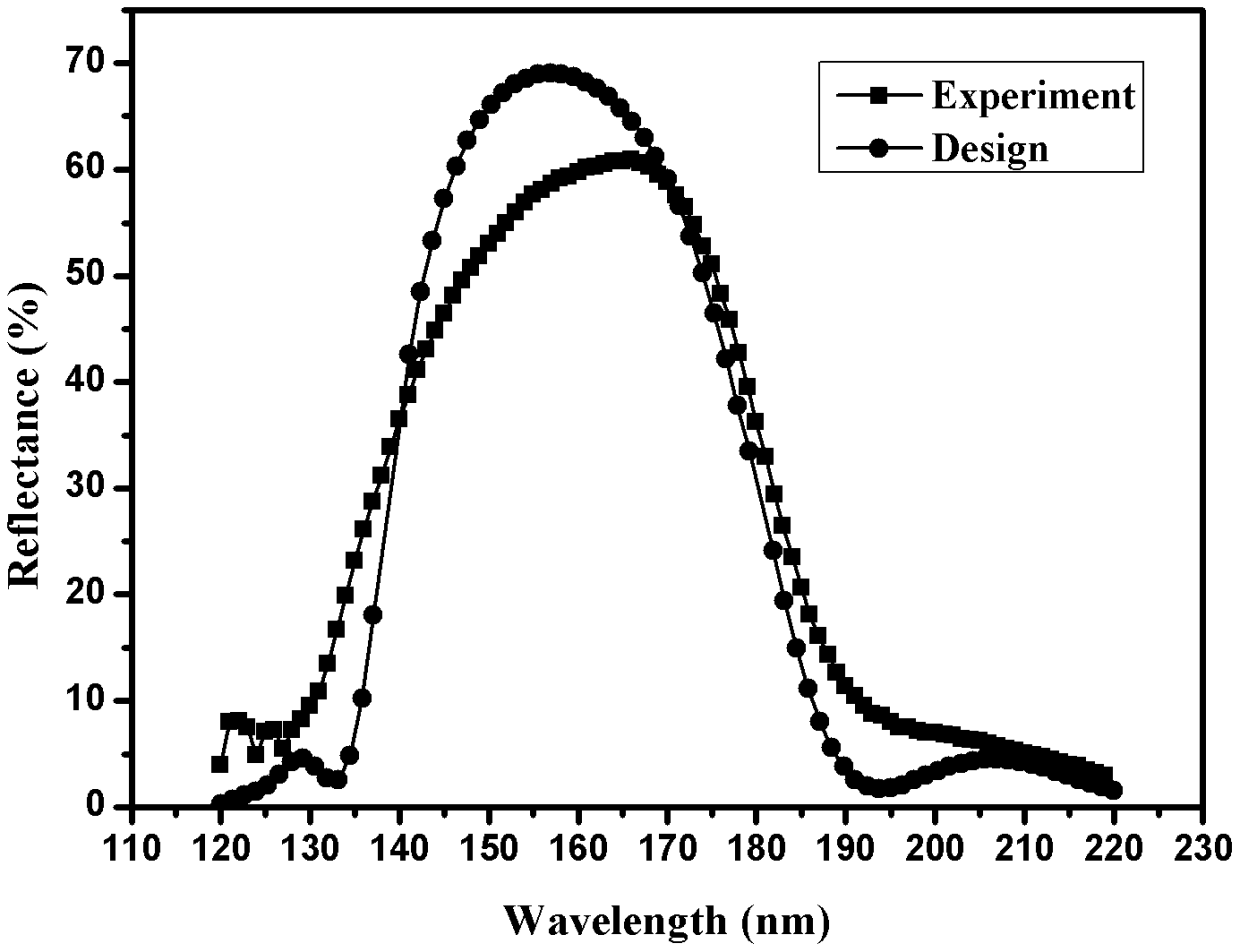
Reflectance curves of designed and fabricated 9-layer non-periodic Al/MgF_2_ multilayer with an incident angle of 22°.

Figure 4 shows reflectance of fabricated 9-layer non-periodic Al/MgF_2_ multilayer, for comparison, and the design curve is also provided. High reflectance in the working wavelength region and better reflectance suppression in unwanted wavelength region are obtained. However, obvious discrepancies between the experiment and theoretical design still exist. This disagreement can be ascribed to film inhomogeneity, surface roughness, and thickness control error, which are not taken into account in theoretical calculation.

**Figure 5:**
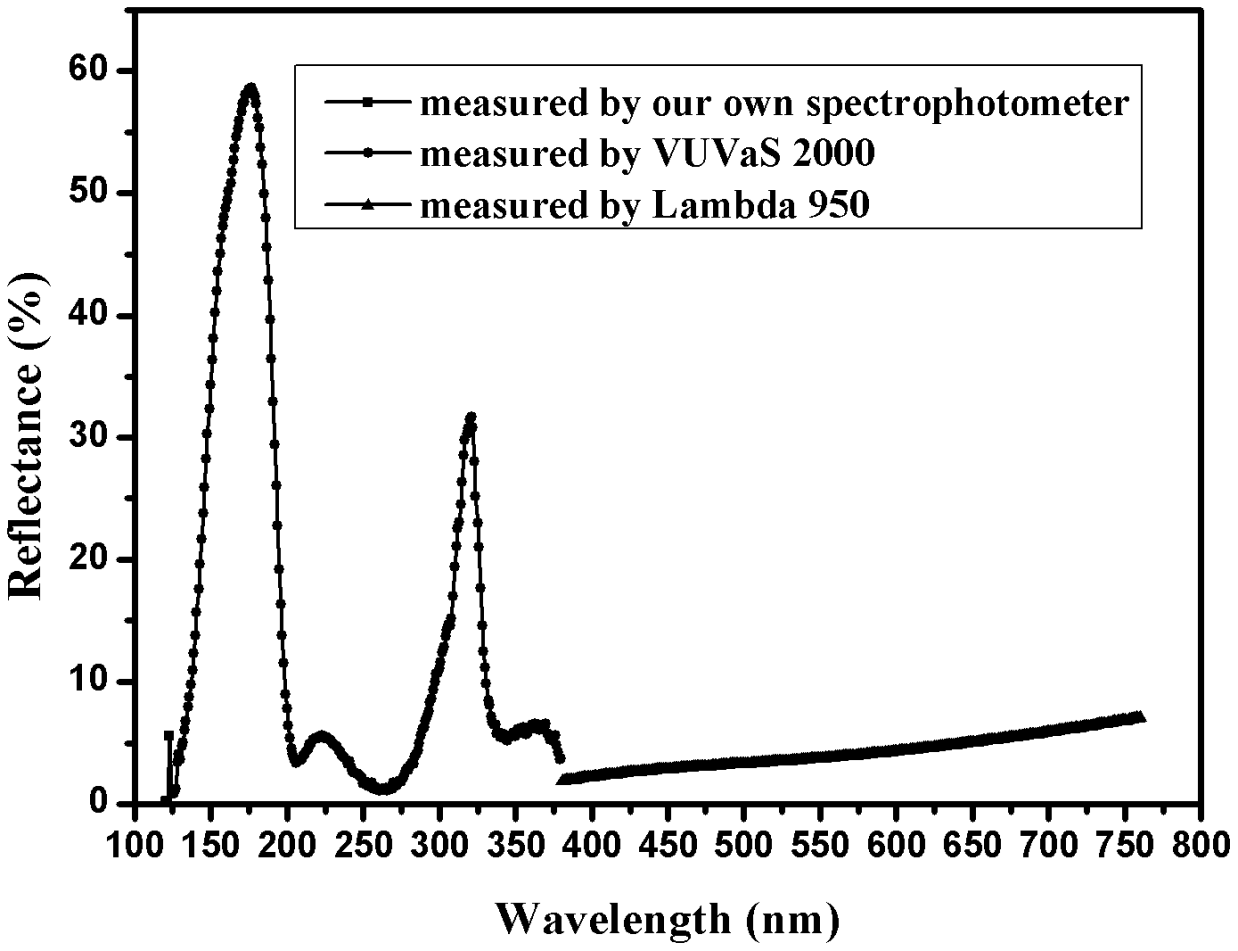
Reflectance curves of fabricated 9-layer non-periodic Al/MgF_2_ multilayer with an incident angle of 8°.

Figure 5 demonstrates a reflectance curve of deposited 9-layer non-periodic Al/MgF_2_ multilayer with an incident angle of 8°. The reflectance of deposited broadband filter in 120–125 nm was measured by our own developed spectrophotometer. The reflectance of deposited broadband filter in 126–380 nm was measured by McPherson VUVaS ultraviolet spectrophotometer, and the reflectance in 381–760 nm was characterized by Lambda 950 UV/VIS/NIR Spectrophotometer. As shown in Figure 5, this broadband filter also has a good reflectance blocking of visible light, and the only drawback is high reflectance of 32% centered at 321 nm.

**Figure 6:**
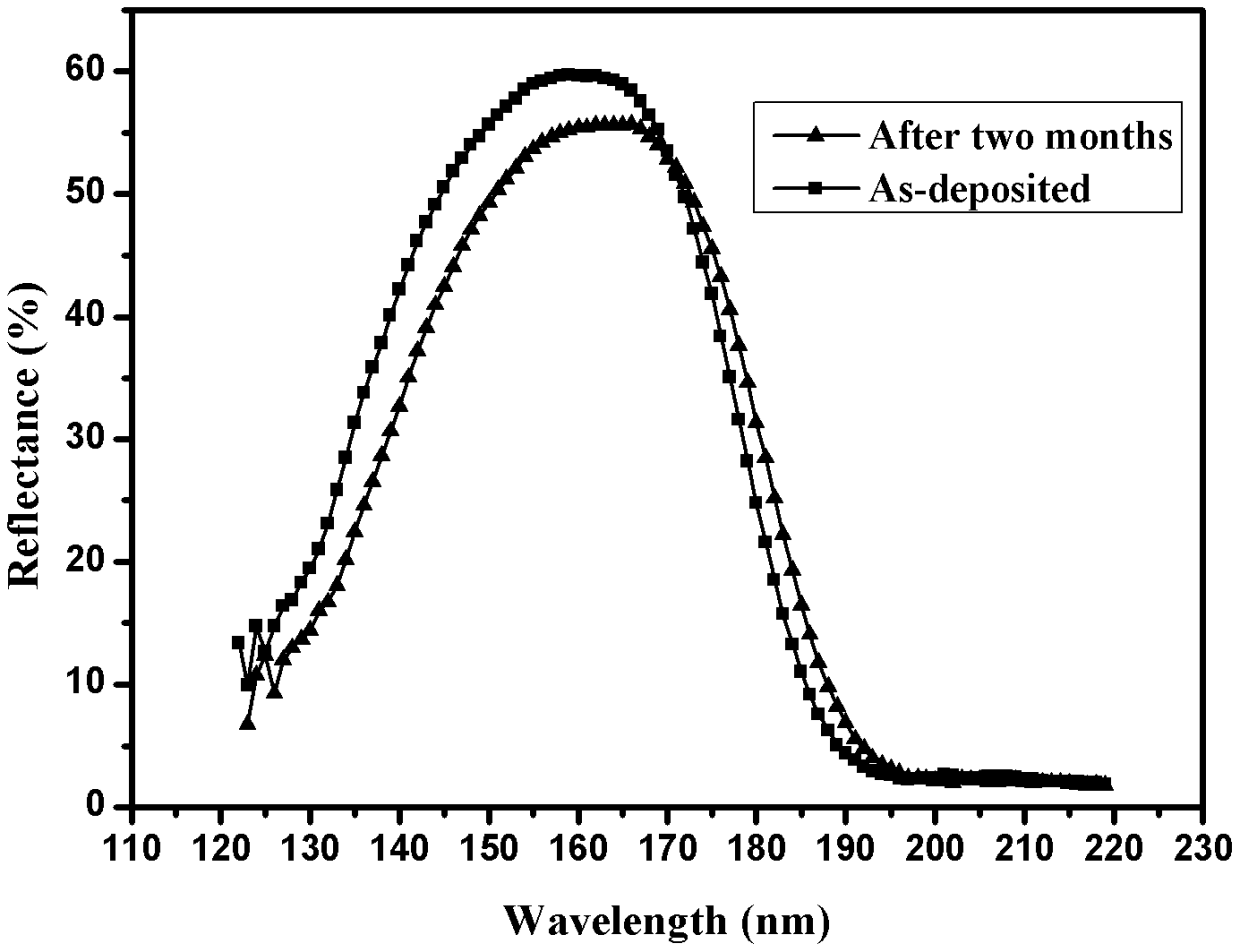
Reflectance curves of as-deposited and two-month aged of 9-layer non-periodic Al/MgF_2_ multilayer with an incident angle of 22°.

Figure 6 shows reflectance curves of as-deposited and two-month aged of 9-layer non-periodic Al/MgF_2_ multilayer, and the incident angle is 22°. The deposited sample was kept in a desiccator at room temperature. After two months, reflectance curve shifts toward longer wavelength, and there is a slight drop of maximum reflectance. Similar phenomenon of Al/MgF_2_ transmittance filters was reported by Bates and Bradley^13^. This change may be explained by absorption of water in MgF_2_ layer^29^ and slow continuing oxidation of Al films^13^. When Al is deposited, little oxygen still exists due to gas release from deposition materials although the base pressure of chamber is 1.3 × 10^−4^ Pa, and reacts with Al films. Oxidation of Al film is a long process, and it can continue after deposition of Al films^26^,^27^,^28^. Moreover, formed oxide layers undergo gradual structural transformation from amorphous phase to crystalline one^28^. In addition, it is generally accepted that MgF_2_ film should be deposited at a high substrate temperature^29^, and Al film should be prepared at room temperature. In order to obtain high reflectance of filters, we do not make the substrate heated. Thus, our MgF_2_ layer is not dense enough, and they maybe undergo slight structural change after deposition. The reason for the shift of reflectance curve is under further investigation.

In Summary, we extend π-multilayer technology to design broadband FUV filters with high-*k* materials, and discuss optical properties of 9-layer Al/MgF_2_ multilayer for *H*/*L* > 1 instead of *H*/*L* < 1. A 9-layer non-periodic Al/MgF_2_ broadband filter was successfully designed and fabricated to meet our requirements. This extended π-multilayer theory is a powerful, promising technology to be employed in designing FUV filters made of metal-dielectric material pairs.

π-multilayer technology is a method to design filters proposed by Zukic and Torr, and they just demonstrated the discussion for the case of low-*k* materials. Here, we provide its application for the case of high-*k* materials. Thus, applications of π-multilayer technology are extensively extended, and filters with specific reflectance and FWHM can be hopefully designed by tuning *H*/*L* ratio.

## Methods

The used Al and MgF_2_ have a purity of 99.99%. The depositions were made in an electron beam evaporation vacuum system. The base pressure was 1.3 × 10^−4^ Pa. Al and MgF_2_ in copper crucible were evaporated by electron beam. The voltage of electron gun was fixed to be 10 kV, the thickness of films and deposition rate were controlled by a quartz crystal (IC6, Inficon Company). The distance between the source and substrate is 50 cm, the distance between the quartz crystal and source is 45 cm. The deposition rate of Al and MgF_2_ was 0.3 nm/s. The substrate was not heated.

The reflectance and transmittance of films in the wavelength range of 120–125 nm were measured by our own developed spectrophotometer with a step of 1 nm, and the base pressure was 2 × 10^−4^ Pa. The reflectance and transmittance of films in the wavelength range of 126–380 nm were measured by McPherson VUVaS ultraviolet spectrophotometer with a step of 1 nm, and the base pressure was 4 × 10^−3^ Pa. Vacuum ultraviolet line can be strongly absorbed by atmospheric oxygen, so the FUV spectral measurements must be performed at low pressure. The reflectance of films in the wavelength range of 381–760 nm was characterized by Lambda 950 UV/VIS/NIR Spectrophotometer with a step of 1 nm in ambient atmosphere.

## Author Contributions

X.D.W. designed research, analyzed data and wrote the paper, Bi.C. and H.F.W. wrote the main manuscript text, X.Z., L.P.H., Bo.C., S.J.L., Z.X.C., X.H.Y. and Y.P.L. prepared figures 1–6. F.H. revised the literature. All authors reviewed the manuscript.
